# Trajectories of dietary patterns from pregnancy to 12 years post-pregnancy and associated maternal characteristics: evidence from the Avon Longitudinal Study of Parents and Children

**DOI:** 10.1007/s00394-023-03185-x

**Published:** 2023-06-09

**Authors:** Sonia Pervin, Pauline Emmett, Kate Northstone, Nick Townsend, Yaqoot Fatima, M. Mamun Huda, H. David McIntyre, Abdullah Al Mamun

**Affiliations:** 1 Institute for Social Science Research, The University of Queensland, 80 Meiers Road, Long Pocket Precinct, Indooroopilly, Brisbane, QLD 4068 Australia; 2 ARC Centre of Excellence for Children and Families Over the Life Course, The University of Queensland, Brisbane, Australia; 3 Centre for Academic Child Health, Population Health Sciences, Bristol Medical School, University of Bristol, Canynge Hall, 39 Whatley Road, Clifton, Bristol, BS8 2PS UK; 4 Population Health Sciences, Bristol Medical School, University of Bristol, Oakfield House, Oakfield Grove, Bristol, BS8 2BN UK; 5 Centre for Exercise, Nutrition and Health Sciences, School for Policy Studies, University of Bristol, 8 Priory Rd, Bristol, BS8 1TZ UK; 6 Poche Centre for Indigenous Health, Faculty of Health and Behavioural Sciences, The University of Queensland, 74 High St, Toowong, QLD 4066 Brisbane, Australia; 7 Centre for Rural and Remote Health, James Cook University, Mount Isa, Queensland Australia; 8 Mater Clinical Unit and Mater Research, Faculty of Medicine, The University of Queensland, Raymond Terrace, South Brisbane, Queensland 4101 Australia

**Keywords:** Dietary patterns, Pregnancy, Dietary patterns trajectories, PCA, Group-based trajectory modelling, Smoking cessation, ALSPAC

## Abstract

**Purpose:**

Dietary patterns (DPs) during pregnancy have been well researched. However, little is known about maternal diet after pregnancy. The aim of the study was to explore maternal DPs longitudinally, examine trajectories over 12 years after pregnancy and identify associated factors.

**Methods:**

Of 14,541 pregnant women enrolled in the Avon Longitudinal Study of Parents and Children (ALSPAC) complete dietary information was available for 5336 women. Principal components analysis (PCA) was used to derive DPs. DP scores at each time point were used to create DP trajectories using group-based trajectory modelling (GBTM). Multinomial logistic regression assessed the association with maternal factors.

**Results:**

A total of six distinct DPs were identified over time with different numbers of DPs at each time point. The “healthy” and “processed” DPs persisted over the 12-year post-pregnancy. Three trajectories of “healthy” and “processed” DPs were identified from GBTM. Half the women were on the moderately healthy DP trajectory with 37% on the lower trajectory and 9% on the higher healthy DP trajectory. 59% of women were on the lower processed DP trajectory with 38% on the moderate trajectory and 3.3% on the higher processed DP trajectory. Low educational attainment, low social class and smoking in pregnancy were independently associated with being on a less favourable DP trajectory over the 12 years.

**Conclusion:**

Health professionals should provide support on smoking cessation along with healthy eating advice during ante-natal counselling. Continued support on eating healthily after pregnancy would be beneficial for mothers and families.

**Supplementary Information:**

The online version contains supplementary material available at 10.1007/s00394-023-03185-x.

## Background

Diet is an important modifiable risk behaviour that has significant links with a range of non-communicable diseases (NCDs) and related health conditions, including obesity [1]; type 2 diabetes [2]; metabolic syndrome [3]; atherosclerosis [4]; carotid artery stenosis [5]; and cancer [6]. Dietary choices that include whole grains, plenty of fruits and vegetables, unsaturated fats and low-fat dairy products and which limit sugar and salt, reduce the risk of all types of NCDs throughout the life-course [7]. The intake of a healthy, energy-balanced diet during pregnancy is crucial to achieving recommended gestational weight gain and reducing adverse pregnancy outcomes [8]. Additionally, the risk of developing long-term, adverse health outcomes such as obesity, high cholesterol levels and high blood sugar levels may increase in women after pregnancy [9]. It is therefore very important for women to eat a healthy diet during pregnancy and continue with this after delivery.

Much research has focussed on diet during pregnancy and its effects on offspring’s health [10–13], however, effect of maternal post-pregnancy diet has received less attention. Maternal healthy dietary intake after pregnancy is critical for breastfeeding newborn babies for the first 4–6 months of life and suppling key nutrients to the newborn through breastmilk [14]. Eating healthy following pregnancy and long-term health benefit in breastfed infants and future wellbeing of women including their reproductive health has been reported, but research on nutrition of women after delivery and metabolic programming outcome of their infants in later life is scarce [14–16]. Despite a few studies on maternal dietary intake after pregnancy, longitudinal studies on post-pregnancy dietary changes and stability have received very little attention [17, 18], highlighting a significant research gaps regarding the topic.

Changes in dietary habits among women from before to during pregnancy have been systematically examined [19]. Suggesting short-term changes in a few food items may occur, including an increased intake of fruits and vegetables, a decrease in fried and fast-foods, egg, tea and coffee consumption without any intervention [19]. A systematic review by Lee et al. evaluating changes and stability in maternal diet during and immediately after pregnancy reported mixed findings for changes in energy and micronutrient intakes [20]. Findings from these 17 studies suggested significant decreases in fruit and vegetable consumption, diet quality and healthy dietary patterns during the transition from pregnancy to early post-pregnancy, alongside increases in processed foods and fat intakes [20]. However, most of the studies included in the review had short follow-up durations from pregnancy to 6 months post-pregnancy, thus more longitudinal research on tracking of dietary patterns from pregnancy to post-pregnancy was recommended.

In the review of Lee et al., there were two studies that had analysed DPs longitudinally from pregnancy to 5 years post-pregnancy [17, 21] and three studies had tracked dietary patterns from pregnancy to post-pregnancy [17, 22, 23]. Out of two longitudinal studies, one study reported changes in energy and micronutrient intakes [21] and other study reported both stability for four DPs (“health conscious,” “processed,” “confectionery,” and “vegetarian”) and changes in DPs (“traditional”) that did not observed at 4 years post-pregnancy [17]. Overall, the review finding for the tracking of DPs suggested that maternal dietary patterns were stable and did not change significantly from pregnancy to post-pregnancy [17, 22, 23]. In addition, a recent study by Dalrymple et al. examined dietary trajectories longitudinally using diet quality index (DQI) from preconception to mid-childhood among mothers and children and reported stable diet quality trajectories across the early life of the child [24]. The study also reported that poorer dietary trajectory was associated with lower maternal age, education, higher maternal pre-pregnancy BMI, multiparity and smoking [24].

In the general population, some studies have investigated DPs longitudinally examining their stability over time [25, 26], and their impact on health-related risk factors and chronic diseases [27–30]. For example one study reported a sharp increase in a fast food based “modern” DP over two decades and its positive associations with cardio-metabolic risks in an adult population in China [30]. Group-based trajectory modelling (GBTM) has been undertaken to explore long-term changes and stability in DPs among different populations and to identify trajectories of DPs over time [27, 29, 31–33]. However, this method has not been used widely to track maternal dietary trajectories from pregnancy into later life [24]. There is a clear research gap in assessing maternal DPs longitudinally and following trajectories of diet into later life. Therefore, the aim of this study was to (1) extend the duration of follow up of maternal DPs to 12 years post-pregnancy, (2) identify trajectories of dietary patterns over time and (3) examine the maternal factors associated with dietary trajectories using data from the Avon Longitudinal Study of Parents and Children (ALSPAC).

## Methods

### Study design and participants

Avon Longitudinal Study of Parents and Children (ALSPAC) is a population-based, prospective, longitudinal British cohort of pregnant women, their partners and offspring established in the 1990s to investigate the development of health and potential diseases from the time of pregnancy in the women to the adulthood of offspring [34]. Pregnant women residing in three health districts of the county of Avon, around Bristol, in southwest England with an estimated date of delivery between April 1, 1991 and December 31, 1992, were eligible and invited to join the study [34]. A cohort of 14,541 pregnancies was established and 13,988 infants survived to 1 year of age [35, 36]. Full details of the study have been described elsewhere [37, 38]. The study had the approval of the ALSPAC Law and Ethics Committee and Local Research Ethics Committees.

### Dietary assessment

Food frequency questionnaires (FFQ) were used to collect dietary data from the women at 32 weeks of pregnancy and then at 4, 8 and 12 years post-pregnancy [39]. Full details of the dietary data assessment and analysis have been reported elsewhere [17, 40]. Briefly, each questionnaire was posted to the women for self-completion at home [41]. At each time point, they were asked to report their current consumption of a wide variety of food and drink types listed in the FFQ using categorical frequencies to indicate how often ‘nowadays’ they consumed each food: (i) never or rarely; (ii) once in 2 weeks; (iii) 1–3 times a week; (iv) 4–7 times a week; (v) more than once a day. More detailed questions were asked about daily intakes of basic foods such as bread (the number of slices per day), low calorie drinks, cola, tea and coffee (the number of cups). The type of milk (full-fat, semi-skimmed, other), bread (white, wholemeal, other) and fat spreads (butter, margarine, other) usually consumed were also recorded.

The data on frequency of consumption were numerically transformed into times consumed per week, to give a quantitative meaning to the frequency categories, as follows: (i) 0; (ii) 0.5; (iii) 2; (iv) 5.5 (v) 10 times per week. Daily foods were converted to times per week and recoded accordingly to keep similar quantitative values. All variables used in the analysis were standardised by subtracting the mean and dividing by the standard deviation for each variable [42]. The questionnaires were modified slightly over time due to changes in the availability of various foods [17]. Separate categories were created at later ages for coated poultry and fish products, vegetarian pies and tuna, which had previously been included in other categories. Therefore, as time progressed, additional foods and drinks were included in the analysis. Before entry into the PCA, some food groups were combined and a detailed list of food groups is presented in the Supplementary File in Table 1. Thus, the number of food items included in the analysis varied by time points e.g. pregnancy (43 items), 4 years (51 items), 8 years (49 items) and 12 years post-pregnancy (48 items).

### Maternal diet from pregnancy to post-pregnancy 12-years

Maternal dietary patterns at 32 weeks of gestation and at 4 years of child’s age have been previously identified in this cohort using principal component analysis (PCA) with varimax rotation. Detail of the identified dietary patterns has been described elsewhere [17, 42]. In brief, five DPs were identified in pregnancy and four DPs at 4 years post-pregnancy. The DPs identified had high positive loading on the following foods and were labelled accordingly: ‘‘Health conscious/healthy’’: salad, fresh fruit, rice, pasta, fish, pulses and non-white bread. ‘‘Traditional’’: all types of vegetables and some items of poultry and red meat. ‘‘Processed’’: white bread, meat pies, sausages, burgers, roasted potatoes, chips, crisps, baked beans. ‘‘Vegetarian’’: meat substitutes, pulses, nuts, herbal tea, with high negative loadings on all meats. ‘‘Confectionery’’: chocolate, sweets, biscuits, cakes, puddings. The traditional dietary pattern component was not extracted at 4-years post-pregnancy [17]. The mothers completed two additional FFQ at 8- and 12-years post-pregnancy. These FFQs were again administered via self-completion questionnaires and an identical analytical technique to that used for the pregnancy and 4-years data was applied. This was PCA with varimax rotation used to analyse standardised food items, described in detail elsewhere [42].

### Covariates

Covariates were selected based on previous relevant studies; they were obtained from self-completed postal questionnaires sent during pregnancy [42–44]. These include maternal social characteristics at the time of pregnancy i.e. maternal age, educational attainment, social class, marital status and ethnicity, maternal behavioural characteristics i.e. smoking and alcohol intake; and perinatal characteristics i.e. number of previous pregnancies (parity), and pre-pregnancy body mass index (BMI). Maternal educational attainment was assessed in three categories, Low: (None/Vocational or < O level), Medium: O level (school certificate obtained at age 16 years) and High: (A level (examination obtained aged at 18 years) or degree or above). Social class was categorised according to maternal occupation using standard UK classifications of occupation including class I (highest), II, III-non-manual, III-manual, IV and V (lowest) [45] and grouped into three categories (high, middle and low social class). Employment status was categorised as “unemployed” or “employed”; women were classified as “unmarried” or “ever married”, and ethnic group was dichotomised into “white” and “non-white”.

Mothers were asked to report alcohol consumption before their current pregnancy by questionnaire at 18 weeks of gestation as: never, < 1 glass/week, at least 1 glass/week, 1–2 glasses/day, at least 3–9 glasses/day and at least 10 glasses/day. The total glasses/week or day were grouped (never/ < 1 glass/week, 1 + glass/week and 1 or more glasses/day) [46]. Women were categorised into non-smokers, light smokers (less than 10 cigarettes per day) and heavy smokers (more than 10 cigarettes per day) based on their report at 32 weeks of pregnancy [47]. Maternal pre-pregnancy weight and height were self-reported and BMI was calculated as weight (kg)/height (m^2^). Pre-pregnancy BMI was categorised according to the World Health Organization as “underweight (< 18.5 kg/m^2^)”, “normal (18.5 and < 25 kg/m^2^)”, “overweight (> 25.0 and < 30 kg/m^2^),” and “obesity (≥ 30 kg/m^2^)” [48].

### Statistical analysis

Using the DP scores constructed at each time point, group-based trajectory modelling (GBTM) was applied to evaluate trajectories of DPs [49]. This method identifies distinct groups of DPs over the four time points [50]. In brief, GBTM is a latent class growth model (LCGM) designed to identify subgroups within the study population that share a similar developmental trajectory for an outcome over time; that means, grouping individuals following analogous trajectories on specific DPs over time [50]. This study modelled DPs trajectories among mothers who had information at all four time points for the two dietary patterns which persisted; “healthy” and “processed”. First, a base model of trajectory was constructed to determine the number of groups, the order of the polynomial functions and group membership of the participants at each time point. Then, model fitting and inference were carried out via GBMT with the “Traj” plugin command in Stata [51] which implements a Newton–Raphson optimization algorithm for maximum likelihood estimation [52]. The model selection exercise was based on the Bayesian information criterion (BIC) and polynomial order.

A censored normal distribution appropriate to continuous data was used to generate representative trajectory curves that best fit the distinct trajectory groups for the overall study population [49]. It cannot be assumed in GBTM that all trajectories follow the same longitudinal changes in DPs, therefore, the shape of trajectory can be modelled as either intercept, linear, quadratic and cubic during model fitting exercise [24]. The number of trajectory groups and the polynomial order, which determines the shape of each trajectory, were selected based on several criteria, including: (i) the model’s BIC, (ii) the statistical significance of the polynomial coefficients; (iii) the sample size in each group; (iv) a close correspondence between the estimated probability of group membership presented as an average posterior probability (> 0.7) and the odds of correct classification (> 5) for all groups [50, 53]. A three-group model with polynomial distribution satisfied the theoretical and statistical criteria and was selected for inclusion in the main model. Output from the polynomial distributions is presented in the Supplementary file Table S1 and Table S2. Two separate trajectories were constructed one for each DP.

The distribution of maternal characteristics (collected during pregnancy) were presented as frequencies and percentages and the distribution of categorical variables were compared using chi-squared tests. Multinomial logistic regression was used to examine the relationship between these mutually adjusted characteristics and membership of the dietary trajectories. The likelihood of a woman following a specific DP trajectory in relation to maternal characteristics, was expressed using relative risk ratios (RRR). The characteristics investigated were maternal age, education, employment status, marital status, social class, ethnicity, parity, pre-pregnancy BMI, smoking and alcohol behaviours. All statistical analyses were carried out using Stata/SE (STATA, Version 16.0, StataCorp, College Station, TX, USA).

## Results

A total of 12,191 women had dietary data at 32 weeks of gestation, 9598 women at 4 years, 7822 women at 8 years and 6932 women at 12 years post-pregnancy. Complete data at all four time points was available for 5336 women. Table 1 presents the baseline socio-demographic, behavioural and perinatal characteristics of the women at 32 weeks of gestation for those with and without complete data. The women included in the analysis were more likely to have higher educational attainment, belong to a higher social class and have been older at the birth of the child than those excluded (Table 1). They were more likely to be employed, married, of white ethnicity, be non-smokers, consume alcohol, have a BMI within the normal range and be experiencing their first pregnancy.

**Table 1:** Baseline characteristics of women during pregnancy in those with and without complete dietary data at all 4 time points in ALSPAC

Maternal characteristics	Total (*N* = 14,541) *	Complete dietary data (*n* = 5336)^1^	Incomplete dietary data (*n* = 9205)^2^	*p* value
Maternal and family characteristics [*n* (%)]				
Maternal age				≤ 0.001
< = 20 years	1011 (7.2)	116 (2.2)	895 (10.3)	
21–30 years	8830 (62.8)	3167 (59.3)	5663 (64.9)	
> = 30 years	4218 (30.0)	2053 (38.5)	2165 (24.8)	
Maternal education				≤ 0.001
CSE/none/vocational	2976 (25.5)	876 (16.9)	2100 (32.2)	
O level	4317 (36.9)	1862 (36.0)	2455 (37.7)	
A level & higher	4397 (37.6)	2434 (47.1)	1963 (30.1)	
Employment status				
Unemployed	4554 (38.9)	1580 (31.7)	2974 (44.3)	
Employed	7143 (61.1)	3404 (68.3)	3739 (55.7)	
Marital status				≤ 0.001
Never married	2572 (19.1)	617 (11.6)	1955 (23.9)	
Ever married	10,913 (80.9)	4679 (88.4)	6234 (76.1)	
Social class				
High social class	3502 (31.6)	1850 (38.2)	1652 (26.5)	≤ 0.001
Middle social class	5168 (46.7)	2229 (46.1)	2939 (47.2)	
Low social class	2403 (21.7)	761 (15.7)	1642 (26.3)	
Ethnic group				≤ 0.001
White	12,047 (97.4)	5247 (98.7)	6800 (96.4)	
Non-white	324 (2.6)	69 (1.3)	255 (3.6)	
Maternal behavioural characteristics				
Smoking status				≤ 0.001
Non-smoker	8951 (78.6)	4362 (86.7)	4589 (72.3)	
Light smoker (1–9 cig/day)	933 (8.2)	302 (6.0)	631 (9.9)	
Heavy smoker (10 + cig/day)	1500 (13.2)	368 (7.3)	1132 (17.8)	
Regular alcohol drinking pattern				≤ 0.001
None/ < 1 glass/week	6079 (46.2)	2248 (42.4)	3831 (48.7)	
1 + glasses/week	5621 (42.7)	2417 (45.6)	398 (40.7)	
1 + glasses/day	1467 (11.1)	632 (12.0)	640 (10.6)	
Pre-pregnancy and perinatal characteristics				
Parity				≤ 0.001
None	5794 (45.0)	2477 (47.8)	3317 (43.1)	
One	4491 (34.9)	1879 (36.2)	2612 (34.0)	
Two	1829 (14.2)	633 (12.2)	1196 (15.6)	
Three and more	760 (5.9)	199 (3.8)	561 (7.3)	
Pre-pregnancy BMI				≤ 0.001
Normal	9235 (79.5)	4056 (80.9)	5179 (78.3)	
Overweight	1747 (15.0)	720 (14.4)	1027 (15.6)	
Obesity	639 (5.5)	234 (4.7)	405 (6.1)	

*Number of women recruited to the ALSPAC study

^1^Women with complete dietary data information at all four time points and included in the analysis

^2^Women with incomplete dietary data missing at least one time point and excluded from the analysis

Three dietary patterns were chosen to best describe the DPs of mothers at 8 and 12 years post-pregnancy compared to 5 and 4 DPs extracted in pregnancy and 4 years post-pregnancy, respectively [17, 42]. More details of the DPs identified in this study are shown in Supplementary Table S3. Two DPs very similar to those in pregnancy were extracted at both 8 and 12 years. At all four time points from pregnancy to post-pregnancy, the first component loaded highly on brown/wholemeal bread, rice, pasta, fresh fruit, salad, fruit juice, fish, poultry, cheese, pulses, plain potatoes, leafy green and other vegetables, carrots, root vegetables, fresh fruits and whole grain breakfast cereal and negatively on white bread and was labelled as “healthy”. The “processed” pattern was identified as third or second components during pregnancy and later time points. This pattern had high factor loadings for white bread, coated poultry products, breaded and battered white fish, biscuits, baked beans, puddings, ice-cream, cakes/buns, meat pies, sausages, pizza, eggs, chips and roast potatoes, sweets, chocolate and fried foods. These two dietary patterns persistently emerged across all time points for women after pregnancy. A new pattern was observed at both later ages, labelled as “high meat”, it had high factor loadings for poultry, red meats, cold meats, coated poultry products and meat pies. The “confectionery”, “traditional”, and “vegetarian” patterns observed at previous timepoints were not evident at later ages.

The correlations between the DP scores at each time point are summarized in Table 2. The “healthy” dietary pattern correlated relatively strongly across all time points, the correlation between pregnancy and 8 years being the lowest (0.34) and between 8 and 12 years being the highest (0.61). The “processed” DPs showed slightly lower correlation coefficients over time ranging from 0.22 between pregnancy and 12 years to 0.57 between 4 and 8 years. For the most part the “healthy” and “processed” DPs were either uncorrelated or very weakly negatively correlated with each other at different time points, however the “healthy” DP in pregnancy showed moderate negative correlations with the “processed” DP at 4 (− 0.31) and 8 (− 0.27) years post-pregnancy. The four-remaining patterns were not consistently derived and showed various correlations, the most notable being negative correlations between the “vegetarian” DPs at the first two time points and the “high meat” DP at the fourth (− 0.41 and − 0.48, respectively).

**Table 2:** Pearson’s correlation coefficients for dietary pattern (DP) scores of 5336 women obtained by PCA at different time points (at pregnancy, 4, 8/9, and 12 years post-pregnancy) in ALSPAC

Dietary patterns	Pregnancy (32 weeks of gestation)					Post-pregnancy (4 years)				Post-pregnancy (8/9 years)			Post-pregnancy (12 years)		
	Healthy	Traditi	Process	Confec	Vege	Healthy	Process	Confec	Vege	Healthy	Process	High meat	Healthy	Process	High meat
Pregnancy															
Healthy	1														
Traditional	− 0.04*	1													
Processed	− 0.05*	− 0.03	1												
Confectionary	− 0.01	− 0.01	− 0.02	1											
Vegetarian	0.01	0.01	− 0.06*	− 0.01	1										
Post-pregnancy (4 years)															
Healthy	0.42*	0.33*	− 0.10*	− 0.08*	0.06*	1									
Processed	− 0.31**	0.09**	0.42**	0.05*	− 0.09**	0.00	1								
Confectionary	0.14**	− 0.05*	0.03^£^	0.42**	− 0.05*	− 0.00	0.01	1							
Vegetarian	0.15**	− 0.10**	− 0.02	− 0.05*	0.51**	0.02	− 0.01	− 0.01	1						
Post-pregnancy (8 years)															
Healthy	0.34**	0.34**	− 0.12**	− 0.07**	0.05*	0.57**	− 0.10**	− 0.04^£^	− 0.01	1					
Processed	− 0.27**	0.03^€^	0.38**	0.07**	− 0.08**	− 0.08**	0.57**	0.05*	− 0.04^£^	− 0.06**	1				
High meat	0.16**	− 0.04*	0.01	0.39**	− 0.04^£^	0.01	− 0.03^£^	0.51**	− 0.03^£^	− 0.02	− 0.04^£^	1			
Post-pregnancy (12 years)															
Healthy	0.40**	0.28**	− 0.10**	− 0.05*	0.10**	0.54**	− 0.13**	− 0.01	0.08**	0.61**	− 0.11**	0.05*	1		
Processed	− 0.04*	− 0.01	0.22**	0.31**	0.01	− 0.05*	0.31**	0.40**	0.04^£^	− 0.09**	0.39**	0.44**	− 0.00	1	
High meat	− 0.20**	0.10**	0.19**	− 0.02	− 0.41**	− 0.05*	0.27**	− 0.05**	− 0.48**	− 0.00	0.26**	− 0.07**	− 0.02	− 0.00	1

Traditi: traditional, Process: processed, Confec: confectionary, Vege: vegetarian

*p value < 0.001

**p value < 0.0001

^€^*p* value < 0.05

^£^*p* value < 0.01

**Fig. 1:**
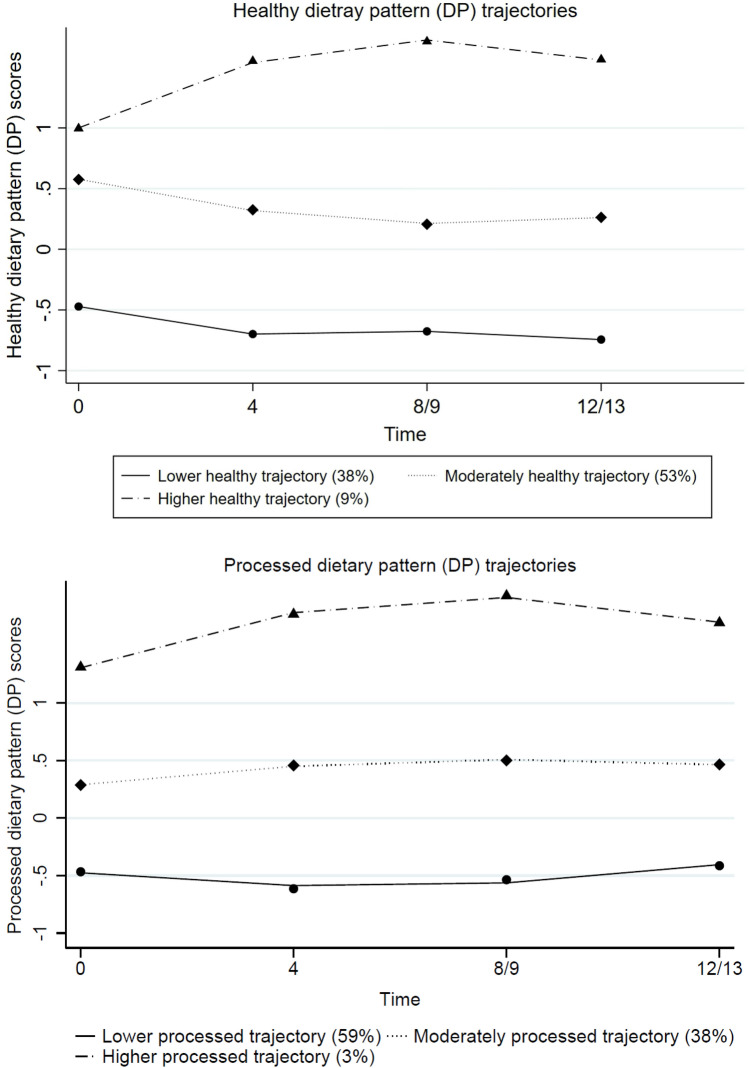
Trajectories of “healthy” and “processed” DPs among mothers over four time points (pregnancy, 4-, 8- and 12-years post pregnancy)

Figure 1 illustrates that women were allocated to three distinct DP trajectories for each of the two consistent DPs (“healthy” and “processed”) over the 12 years since pregnancy. For the healthy DP trajectory, women in Group 1 (*n* = 1971, 37.4%) had an initially negative DP score with a stable progression which remained negative across time (named “lower healthy DP trajectory”); women in Group 2 (*n* = 2918, 53.4%) had a moderate initial DP score with a slow decrease thereafter (named as “moderately healthy DP trajectory”); and, women in Group 3 (*n* = 447, 9.2%) had the highest DP score and increased over the time (named as “higher healthy DP trajectory”). Similarly, women were allocated to one of three trajectory groups for the processed DP: women in Group 1 (*n* = 3171, 58.6%) had a negative DP score initially, with a slight increase thereafter, but it remained negative across the time points (named as “lower processed DP trajectory”); women in Group 2 (*n* = 2011, 38.1%) had an initial moderate score with a slight increase and stable thereafter (named “moderately processed DP trajectory”); however, women in Group 3 (*n* = 154, 3.3%) had the highest processed DP score initially and rapidly increased over the time (labelled as “higher processed DP trajectory”). The distribution of maternal characteristics in women following the “healthy” and “processed” DP trajectories are presented in the Supplementary file Tables S4 and S5.

The associations between maternal baseline characteristics and DP trajectories are presented in Tables 3 and 4. For the healthy DP trajectories women following the ‘higher healthy DP trajectory’ were the reference group. In the fully adjusted models, women on the ‘lower healthy DP trajectory’ were more likely to be from the middle or low social class, with medium or low educational attainment and have smoked heavily in pregnancy and their child was less likely to have been first born compared to women on the ‘higher healthy DP trajectory’. Maternal characteristics did not differ between the ‘higher’ and ‘moderately’ healthy DP trajectories in the adjusted model. For the processed DPs, the comparisons were with women following the ‘lower processed DP trajectory’. Women who followed the ‘moderately processed DP trajectory’ were more likely to be in of middle or low social class, have low educational attainment and having been exposed to obesity prior to pregnancy. Additionally, these women were slightly more like to have been light smokers and more likely to have been aged over 20 years or to have been regular alcohol drinkers compared to women in the reference trajectory. Again, women in the ‘higher processed DP trajectory’ were more likely to have a medium or low educational attainment, to have been a heavy smoker during pregnancy and were slightly more likely to be in the middle social class compared to women on the ‘low processed DP trajectory’. However, they were less likely to have been aged over 30 years when pregnant.

**Table 3:** Multinomial logistic regression analysis on the association of maternal baseline characteristics and healthy dietary patterns trajectories of 5336 women in the ALSPAC study

	Unadjusted model					Adjusted model^b^				
Maternal characteristics^a^	Group 3: Higher healthy DP trajectory^3^	Group 2: Moderately healthy DP trajectory^2^		Group 1: Lower healthy DP trajectory^1^		Group 3: Higher healthy DP trajectory^3^	Group 2: Moderately healthy DP trajectory^2^		Group 1: Lower healthy DP trajectory^1^	
	Ref	RRR (95% CI)	*p* value	RRR (95% CI)	*p* value	Ref	RRR (95% CI)	*p* valve	RRR (95% CI)	*p* value
Maternal age										
< 20 years	1	1		1		1	1		1	
21–30 years		1.38 (0.57–3.34)	0.60	0.45 (0.19–1.05)	0.06		1.56 (0.56–4.36)	0.40	1.02 (0.38–2.75)	0.97
> = 30 years		1.07 (0.44–2.59)	0.66	**0.20 (0.09–0.47)**	** < 0.001**		1.23 (0.43–3.50)	0.69	0.65 (0.24–1.80)	0.41
Maternal education										
A level & higher	1	1		1		1	1		1	
O level		**1.27 (1.01–1.60)**	** < 0.05**	**4.05 (3.17–5.16)**	** < 0.001**		1.16 (0.87–1.55)	0.31	**2.61 (1.92–3.55)**	** < 0.001**
CSE/none/Vocational		1.09 (0.77–1.54)	0.60	**6.90 (4.90–9.71)**	** < 0.001**		0.98 (0.65–1.48)	0.92	**3.98 (2.62–6.04)**	** < 0.001**
Employment status										
Unemployed	1	1		1		1	1		1	
Employed		**1.47 (1.18–1.82)**	** < 0.001**	1.15 (0.92–1.43)	0.22		1.27 (0.97–1.66)	0.08	1.30 (0.98–1.73)	0.07
Marital status										
Never married	1	1		1		1	1		1	
Ever married		1.30 (0.95–1.78)	0.10	0.78 (0.57–1.07)	0.12		1.44 (0.98–2.10)	0.06	1.25 (0.84–1.87)	0.27
Social class										
High social class	1	1				1	1		1	
Middle social class		1.20 (0.95–1.51)	0.11	**3.47 (2.72–4.43)**	** < 0.001**		1.13 (0.86–1.49)	0.38	**1.91 (1.42–2.57)**	** < 0.001**
Low social class		0.84 (0.62–1.14)	0.27	**2.82 (2.05–3.87)**	** < 0.001**		0.86 (0.60–1.24)	0.42	1.43 (0.97–2.10)	0.07
Ethnicity										
White	1	1		1		1	1		1	
Non-white		0.62 (0.30–1.30)	0.21	0.57 (0.26–1.25)	0.16		1.65 (0.50–5.48)	0.41	0.85 (0.22–3.30)	0.80
Smoking status										
Non-smoker	1	1		1		1	1		1	
Light smoker		0.99 (0.62–1.57)	0.96	**1.94 (1.21–3.10)**	** < 0.01**		1.00 (0.59–1.70)	0.99	1.52 (0.88–2.61)	0.13
Heavy smoker		2.03 (0.98–4.19)	0.06	**8.78 (4.31–17.9)**	** < 0.001**		1.81 (0.82–4.01)	0.14	**5.33 (2.44–11.6)**	** < 0.001**
Alcohol intake										
Never/ < 1 glass/week	1	1		1		1	1		1	
1 + glasses/week		1.09 (0.88–1.36)	0.28	**0.68 (0.54–0.84)**	** < 0.01**		1.11 (0.87–1.42)	0.40	0.80 (0.62–1.05)	0.10
1 + glasses/day		1.04 (0.76–1.43)	0.93	**0.55 (0.39–0.77)**	** < 0.01**		1.10 (0.77–1.60)	0.59	0.73 (0.49–1.09)	0.12
Parity										
None	1	1		1		1	1		1	
One		0.80 (0.64–1.01)	0.06	**0.78 (0.62–0.99)**	** < 0.05**		0.81 (0.61–1.07)	0.14	**0.70 (0.52–0.95)**	** < 0.05**
Two		**0.63 (0.46–0.86)**	** < 0.01**	0.79 (0.58–1.09)	0.16		0.65 (0.44–0.96)	0.02	0.71 (0.47–1.07)	0.10
3 and more		0.88 (0.50–1.56)	0.68	1.12 (0.63–1.98)	0.70		0.82 (0.44–1.56)	0.55	0.93 (0.47–1.83)	0.83
Pre-pregnancy BMI										
Normal	1	1		1		1	1		1	
Overweight		0.84 (0.63–1.12)	0.25	0.99 (0.74–1.32)	0.96		0.88 (0.64–1.21)	0.42	0.87 (0.62–1.23)	0.43
Obesity		1.13 (0.66–1.93)	0.64	1.46 (0.85–2.52)	0.16		1.32 (0.71–2.45)	0.37	1.17 (0.61–2.23)	0.63

DP dietary pattern

^a^Data are presented as Relative risk ratio (RRR) of following “^1^Group 1: Lower healthy trajectory” and “^2^Group 2: Moderately healthy trajectory” compared with “^3^Group 3: Higher healthy trajectory” as the reference group

^b^All variables are first presented unadjusted and then fully adjusted for all other variables in the table e.g. maternal age, education, employment status, marital status, social class, ethnicity, smoking status, alcohol intake, parity and pre-pregnancy BMI

**Table 4:** Multinomial logistic regression analysis on the association of maternal baseline characteristics and processed dietary trajectories of 5336 women in the ALSPAC study

Maternal characteristics^a^	Unadjusted model					Adjusted model^b^				
	Group 1: Lower processed DP trajectory^3^	Group 2: Moderately processed DP trajectory^2^		Group 3: Higher processed DP trajectory^1^		Group 1: Lower processed DP trajectory^3^	Group 2: Moderately processed DP trajectory^2^		Group 3: Higher processed DP trajectory^1^	
	Ref	RRR (95% CI)	*p* value	RRR (95% CI)	*p* value	Ref	RRR (95% CI)	*p* value	RRR (95% CI)	*p* value
Maternal age										
< 20 years	1	1		1		1	1		1	
21–30 years		**0.34 (0.21–0.55)**	** < 0.001**	**0.29 (0.13–0.64)**	** < 0.01**		**0.27 (0.15–0.49)**	** < 0.001**	0.40 (0.13–1.24)	0.11
> = 30 years		**0.15 (0.09–0.24)**	** < 0.001**	**0.07 (0.03–0.15)**	** < 0.001**		**0.16 (0.09–0.30)**	** < 0.001**	**0.21 (0.06–0.70)**	** < 0.01**
Maternal educational attainment										
A level & higher	1	1		1		1	1		1	
O level		**2.74 (2.40–3.12)**	** < 0.001**	**4.64 (2.89–3.12)**	** < 0.001**		1.83 (1.55–2.15)	< 0.001	**2.84 (1.60–5.06)**	** < 0.001**
CSE/none/vocational		**3.33 (2.82–3.92)**	** < 0.001**	**10.1 (6.22–16.5)**	** < 0.001**		**1.96 (1.58–2.44)**	** < 0.001**	**4.39 (2.33–8.29)**	** < 0.001**
Employment status										
Unemployed	1	1		1		1	1		1	
Employed		**0.74 (0.65–0.84)**	** < 0.001**	**0.46 (0.32–0.65)**	** < 0.001**		0.87 (0.74–1.02)	0.09	0.63 (0.40–1.01)	0.05
Marital status										
Unmarried	1	1		1		1	1		1	
Ever married		**0.77 (0.65–0.92)**	** < 0.01**	**0.42 (0.28–0.63)**	** < 0.001**		1.05 (0.83–1.33)	0.69	0.66 (0.37–1.17)	0.15
Social class										
High social class	1	1		1		1	1		1	
Middle social class		**2.35 (2.05–2.68)**	** < 0.001**	**3.58 (2.25–5.71)**	** < 0.001**		**1.40 (1.19–1.66)**	** < 0.001**	**1.79 (1.01–3.18)**	** < 0.05**
Low social class		**2.69 (2.25–3.22)**	** < 0.001**	**4.98 (2.91–8.53)**	** < 0.001**		**1.58 (1.27–1.96)**	** < 0.001**	1.95 (1.00–3.83)	0.05
Ethnicity										
White	1	1		1		1	1		1	
Non-white		1.40 (0.85–2.29)	0.18	**3.10 (1.20–8.05)**	** < 0.05**		1.11 (0.56–2.19)	0.77	2.58 (0.57–11.6)	0.21
Smoking status										
Non-smoker	1	1		1		1	1		1	
Light smoker		**1.60 (1.26–2.03)**	** < 0.001**	**2.40 (1.34–4.29)**	** < 0.01**		**1.40 (1.05–1.85)**	** < 0.05**	1.61 (0.79–3.27)	0.18
Heavy smoker		**2.18 (1.75–2.72)**	** < 0.001**	**3.87 (2.39–6.25)**	** < 0.001**		1.28 (0.97–1.70)	0.08	**2.01 (1.09–3.69)**	** < 0.05**
Alcohol intake										
Never/ < 1 glass/week	1	1		1		1	1		1	
1 + glasses/week		**0.70 (0.62–0.79)**	** < 0.001**	0.63 (0.45–0.89)	< 0.01		0.84 (0.73–0.97)	< 0.05	0.92 (0.59–1.42)	0.70
1 + glasses/day		**0.46 (0.37–0.56)**	** < 0.001**	0.56 (0.32–0.98)	< 0.05		**0.64 (0.51–0.82)**	** < 0.001**	0.91 (0.46–1.82)	0.80
Parity										
None	1	1		1			1		1	
One		1.07 (0.94–1.21)	0.31	1.14 (0.80–1.66)	0.48	1	1.01 (0.86–1.20)	0.88	0.90 (0.53–1.51)	0.68
Two		**1.19 (1.00–1.43)**	** < 0.05**	1.52 (0.93–2.49)	0.09		1.10 (0.87–1.40)	0.43	1.25 (0.64–2.44)	0.51
Three and more		1.07 (0.79–1.45)	0.65	1.62 (0.76–3.47)	0.21		0.94 (0.63–1.39)	0.74	0.82 (0.23–2.92)	0.76
Pre-pregnancy BMI										
Normal	1	1		1		1	1		1	
Overweight		1.08 (0.91–1.27)	0.34	1.30 (0.82–2.05)	0.26		0.97 (0.80–1.18)	0.79	0.94 (0.53–1.64)	0.81
Obesity		**1.69 (1.29–2.22)**	** < 0.001**	**2.06 (1.05–4.05)**	** < 0.05**		**1.53 (1.11–2.10)**	** < 0.01**	0.89 (0.31–2.53)	0.82

DP dietary pattern

^a^Data are presented as Relative risk ratio (RRR) of following “^2^Group 2: Moderately processed trajectory” and “^1^Group 3: Higher processed trajectory” compared with “^3^Group 1: Lower processed trajectory” as the reference group

^b^All variables are first presented unadjusted and then fully adjusted for all other variables in the table e.g. maternal age, education, employment status, marital status, social class, ethnicity, smoking status, alcohol intake, parity and pre-pregnancy BMI

## Discussion

This study models group-based trajectories of longitudinal dietary patterns among British women from pregnancy across multiple timepoints post-pregnancy. Women’s dietary intakes were collected prospectively in ALSPAC four times from pregnancy to 12 years post-pregnancy. Six different dietary patterns were derived using PCA analysis over the four time points. A “healthy” and a “processed” DP were persistent at each time and used to explore the trajectories of the women’s diets across time. Three trajectories were identified for each of the two patterns.

For the “healthy” DP trajectories, the majority (53.4%) of women followed the “moderately healthy DP trajectory”. This implies that this group of women have a moderate score on the healthy DP at each time point. A further 37.4% of women followed a “lower healthy DP trajectory” where they had a lower score on the healthy DP at each time point. The remaining women (9.2%) were on a “higher healthy trajectory”, representing higher scores on the healthy DP across time. This is the smallest group and we can speculate that these women should have a better health profile than the group who had followed the “lower healthy DP trajectory” throughout the period of interest. Further research will be necessary to confirm this. For the “processed” trajectory, most women followed the “lower processed DP trajectory” (58.6%)”. These women had a lower score on the processed DP throughout. The “moderately processed DP trajectory” (38.1%) was the second largest trajectory group with women having moderate scores on the “processed” DP at all time points. While the “higher processed DP trajectory” was followed by only 3.3% of the women, this trajectory implies the consumption of a highly processed diet at each time point. Further research is necessary to understand the health effects on women who were following the “higher processed DP trajectory” over time.

Of the remaining DPs in pregnancy “confectionary” and “vegetarian” were not derived after 4 years post-pregnancy and the “traditional” DP only occurred in pregnancy. A new DP emerged at 8 years post-pregnancy and continued at 12 years it was named “high meat”. These findings suggest that some DPs emerged in this cohort were not necessarily stable over time. A shift toward a more meat-based diet among women supports the global view of nutritional transition towards animal-based diets which may be linked to increased risk of nutrition related NCDs [54–56]. Further investigation of these unstable patterns is warranted.

Our study found that women were more likely to have consistently low or moderate scores on a DP across time than consistently high scores on that pattern. Only a small number of women who started with a high “healthy” DP score, remained high over time, thus having a “healthy” dietary pattern throughout. This was also true for the “processed” DP trajectories. Women with medium or low educational attainment were more likely to follow the “lower healthy” and “higher processed” DP trajectories in this cohort. This level of stability in dietary trajectories and association with the educational attainment of women, was similar to another British study the Southampton Women’s Survey (SWS) that examined longitudinal changes in dietary quality trajectories in women from pre-pregnancy to 8–9 years post-pregnancy [24]. The stability of DP trajectories identified in our study are also in line with studies reporting dietary shifts over time among Chinese adults, although the identified dietary patterns differed from those in our study [27, 32, 57]. This may suggest that the studies that identified changes in dietary intake from pregnancy to post-pregnancy may be due to short-term changes related to maternal motivation to make changes during pregnancy and while breastfeeding their newborn [20].

A limited number of studies have investigated changes in DPs among adult populations using group-based trajectory modelling over long time periods and diverse dietary trajectories have emerged [27, 28, 32, 57–59]. DP trajectories have been found to vary between studies in relation to the sex of the participants, socioeconomic background, ethnic group and by cultural diversity. Variations in DPs between studies are likely to be due to cultural differences in dietary habits, different analytical approaches used to identify the DPs as well as differences in the number and type of food items included in the analysis [60]. A large variation in DP trajectories has been reported both between and within populations [27, 32]. Thus, earlier studies that have demonstrated divergence in DP trajectories may not be directly comparable with our study [32, 59]. Given the paucity of evidence, more research is needed among women after pregnancy to understand maternal dietary trajectories across the life course.

Compared to women following the “higher healthy DP trajectory”, maternal characteristics in pregnancy did not differ in those following the “moderately healthy DP trajectory”. However, women following the “lower healthy DP trajectory” were more likely to have smoked heavily in pregnancy. A similar difference occurred between women following the “higher processed DP trajectory” compared those following the “lower processed DP trajectory”. These results suggest that smoking in pregnancy was associated with having a less favourable DP at all time points. These findings are consistent with previous studies showing an association between smoking and unhealthy dietary intake among women [24, 61–64]. Again, a study on smoking and dietary intake among Norwegian pregnant women found that women who smoked during pregnancy consumed significantly less healthy foods and more unhealthy foods compared to non-smoking women [62]. Importantly, women who smoked tended to have a less nutritious dietary intake [62], a higher fat and energy intake and increased craving for highly palatable foods [63, 64] compared to non-smokers throughout pregnancy.

There were social class differences between women following the different DP trajectories. Women who belonged to the lower social class had a higher chance of following the “lower healthy DP trajectory” compared to following either the higher or moderately healthy DP trajectories. Furthermore, women with a low or middle social class background were more likely to follow the “moderately” or “higher processed DP trajectory” rather than following the “lower processed DP trajectory”. People with lower social status are likely to have less disposable income and evidence suggests that lower income groups tend to fall into lower quintiles for food expenditure [65]. Another study also found that lower quality diets tended to be consumed by economically disadvantage populations [66]. Our findings align with British SWS cohort which also reported that women following a poor diet quality trajectory were more likely to be in a lower social class [24]. These associations may be due to the high price of healthy foods, limits on budgets for food, constraints of work-life balance making it more difficult to cook from scratch and leading to the purchase of ready-packed foods often of low nutritional quality [32, 65–68].

The major strength of this study is its prospective cohort design, over 12 years of follow-up (from pregnancy to post-pregnancy) and complete dietary information from more than 5000 women. The ALSPAC cohort provided a unique opportunity to explore longitudinal dietary patterns and dietary trajectories because of its large sample size and long follow-up. The use of dietary patterns at 4 time points from pregnancy allowed the assessment of dietary stability over time and the development of dietary trajectories among British women. Additionally, GBMT modelling proved to be an important technique when classifying the dietary patterns into interpretable trajectories. A wide range of potential confounders was available to the study.

Nevertheless, this study has some limitations that need to be recognised. Firstly, data on dietary intake was self-reported and hence may be susceptible to measurement bias and inaccuracies. However, the use of FFQs is well-accepted for dietary assessment in observational nutritional science [69]. Secondly, the number of food items and PCA loading scores were slightly different at each time point, due to slight modifications of the FFQ over time e.g. additional food and drink items [17, 70]. Further, some number of women who completed the pregnancy questionnaire failed to complete follow-up questionnaires thus reducing the size and changing the composition of the cohort. This led to the decision to only use data from women with complete dietary data across four time points in this study while providing Table 1, pointing out the likely biases due to the reduced sample size so that the reader can take them into consideration. The biases in our study were very similar to those in SWS a British cohort study in a different part of the country [24] and the fact that the findings were very similar should provide confidence that they are generalisable in a British context. Although our study identified some unstable DPs in the mothers’ diets, we were unable to explore these fully in this analysis.

## Conclusion

Following pregnancy, continuing with a healthy and optimal diet during post-pregnancy is important to support maternal health and wellbeing and for the child to learn healthy eating practices. In our study, maternal DPs were found to have some variability from pregnancy to later time points, with some evidence of a move towards higher consumption of a meat-based diet. However, within the persistent “healthy” and “processed” DPs, dietary trajectories were relatively stable over the 12 years post-pregnancy. Our findings add to the evidence for strengthening and improving maternal care during pregnancy and for continued counselling to mothers regarding healthy eating and smoking cessation while bringing up their children.

Our findings suggest that it is advisable that health professionals target smoking cessation at the same time as promoting a healthy diet in antenatal care. If resources allowed healthy eating and ante-smoking education that started well before pregnancy may prove even more beneficial to family health. Also, follow up support for healthy eating after the birth of the child which is likely to benefit the mother and her family. To increase effectiveness, introducing a triage system to prioritise socially and economically disadvantaged women with risk behaviours would be beneficial for promoting life course healthy eating among women.

## Supplementary Information

Below is the link to the electronic supplementary material.

Supplementary file1 (DOCX 64 KB)

## Data Availability

ALSPAC data used in this paper are available upon application to the Executive of ALSPAC (alspac-exec@bristol.ac.uk). The ALSPAC study website contains details of all the data that is available through a fully searchable data dictionary and variable search tool (http://www.bristol.ac.uk/alspac/researchers/our-data/). The ALSPAC data management plan and details of the policy regarding data sharing are also publicly available (http://www.bristol.ac.uk/alspac/researchers/dataaccess/documents/alspac-data-management-plan.pdf).
