# Supplementary material for: Trajectories of dietary patterns from pregnancy to 12 years post-pregnancy and associated maternal characteristics: evidence from the Avon Longitudinal Study of Parents and Children
Source: Eur J Nutr. 2023 Jun 9;62(7):2763–77. doi: 10.1007/s00394-023-03185-x (PMC10468914; doi:10.1007/s00394-023-03185-x)
Supplement: Supplementary file 1 — Supplementary file1 (DOCX 64 KB) [file 394_2023_3185_MOESM1_ESM.docx]

**Supplementary document**

**Title: Trajectories of dietary patterns from pregnancy to 12/13 years post-pregnancy and associated maternal factors: evidence from the Avon Longitudinal Study of Parents and Children (ALSPAC)**

**Authors’ name:**

Sonia Pervin,^1, 2*^ Pauline Emmett,^3^ Kate Northstone,^4^ Nick Townsend,^5^ Yaqoot Fatima, ^2,6,7^ M Mamun Huda,^2, 6^ H. David McIntyre,^8^ Abdullah Al Mamun^2, 6^

**Author’s affiliation:**

^1^Institute for Social Science Research, The University of Queensland, Brisbane, Australia.

^2^ARC Centre of Excellence for Children and Families over the Life Course, The University of Queensland, Brisbane, Australia

^3^Centre for Academic Child Health, Population Health Sciences, Bristol Medical School, University of Bristol, Canynge Hall, 39 Whatley Road,, Clifton, Bristol BS8 2PS.

^4^Population Health Sciences, Bristol Medical School, University of Bristol, Oakfield House, Oakfield Grove, Bristol, BS8 2BN.

^5^Centre for Exercise, Nutrition and Health Sciences, School for Policy Studies, University of Bristol, 8 Priory Rd, Bristol BS8 1TZ.

^6^Poche Centre for Indigenous Health, Faculty of Health and Behavioural Sciences, The University of Queensland, 74 High St, Toowong QLD 4066

^7^Centre for Rural and Remote Health, James Cook University, Mount Isa, Queensland, Australia

^8^Mater Clinical Unit and Mater Research, Faculty of Medicine, The University of Queensland, Raymond Terrace, South Brisbane, Queensland, 4101.

*** Corresponding author:**

Sonia Pervin

Institute for Social Science Research

ARC Centre of Excellence for Children and Families over the Life Course

The University of Queensland

80 Meiers Road, Long Pocket Precinct, Indooroopilly, Queensland 4068, Australia

Tel + 61 470653442

Fax: + 61 7 334 67646

E-mail: [s.pervin@uq.edu.au](mailto:s.pervin@uq.edu.au)

Table S1: Selection process for the trajectory groups of healthy dietary patterns

| Number of groups | Trajectory shapes (orders) | BIC | AIC | Proportion of groups (%) | | | |
| --- | --- | --- | --- | --- | --- | --- | --- |
|  |  |  |  | Group 1 | Group 2 | Group 3 | Group 4 |
| 3 | 1 1 1 | -27010.73 | -26981.10 | 36.7 | 53.5 | 9.7 |  |
| 3 | 1 1 2 | -26990.00 | -26957.08 | 37.9 | 53.3 | 8.7 |  |
| 3 | 1 2 2 | -26934.34 | -26898.13 | 37.3 | 53.4 | 9.2 |  |
| 3 | 1 2 1 | -26967.83 | -26934.90 | 36.0 | 53.7 | 10.1 |  |
| 3 | **2 2 2** | **-26928.62** | **-26889.12** | **37.8** | **53.1** | **9.0** |  |
| 3 | 2 2 1 | -26961.98 | -26925.77 | 36.5 | 53.4 | 9.9 |  |
| 3 | 2 1 1 | -26997.86 | -26964.94 | 37.4 | 53.0 | 9.4 |  |
| 3 | 2 1 2 | -26976.35 | -26940.14 | 38.5 | 52.9 | 8.5 |  |
| 3 | 3 2 2 | -26922.94 | -26880.15 | 37.6 | 53.3 | 9.0 |  |
| 3 | 3 1 1 | -26991.63 | -26955.42 | 37.2 | 53.2 | 9.5 |  |
| 3 | 1 1 3 | -26994.10 | -26957.89 | 37.8 | 53.3 | 8.7 |  |
| 4 | 1 1 1 1 | -26837.55 | -26798.04 | 29.1 | 51.5 | 17.7 | 1.5 |
| 4 | 2 2 2 2 | -26764.35 | -26711.68 | 23.6 | 47.1 | 24.8 | 4.4 |
| 4 | 1 2 2 1 | -26781.72 | -26735.64 | 33.0 | 52.6 | 13.5 | 0.8 |
| 4 | 2 1 1 2 | -26804.70 | -26758.62 | 27.2 | 49.2 | 20.6 | 2.8 |
| 4 | 1 1 2 2 | -26781.86 | -26735.77 | 20.6 | 43.7 | 29.7 | 5.8 |
| 4 | 2 2 2 1 | -26794.81 | -26745.43 | 27.2 | 51.0 | 19.7 | 1.8 |

Table S2: Selection process for the trajectory groups of processed dietary patterns

| Number of groups | Trajectory shapes (orders) | BIC | AIC | Proportion of groups (%) | | |
| --- | --- | --- | --- | --- | --- | --- |
|  |  |  |  | Group 1 | Group 2 | Group 3 |
| 2 | 2 2 | -27272.77 | -27246.43 | 69.9 | 30.0 |  |
| 2 | 3 3 | -27276.52 | -27243.60 | 69.9 | 30.0 |  |
| 2 | 1 1 | -27314.47 | -27294.71 | 70.3 | 29.6 |  |
| 3 | 1 1 1 | -27000.25 | -26970.62 | 59.1 | 37.6 | 3.2 |
| 3 | 1 1 2 | -26987.87 | -26954.95 | 59.1 | 37.5 | 3.3 |
| 3 | 1 2 2 | -26985.85 | -26949.64 | 59.5 | 37.3 | 3.1 |
| 3 | 1 2 1 | -26994.07 | -26961.14 | 59.6 | 37.2 | 3.1 |
| **3** | **2 2 2** | **-26947.92** | **-26908.41** | **58.6** | **38.0** | **3.3** |
| 3 | 2 2 1 | -26954.37 | -26918.15 | 58.5 | 37.9 | 3.4 |
| 3 | 2 1 1 | -26969.65 | -26936.72 | 58.0 | 38.4 | 3.5 |
| 3 | 2 1 2 | -26957.32 | -26921.11 | 58.0 | 38.4 | 3.5 |
| 3 | 3 1 1 | -26968.66 | -26932.45 | 57.9 | 38.4 | 3.5 |
| 3 | 3 1 2 | -26956.29 | -26916.79 | 58.0 | 38.4 | 3.5 |
| 3 | 3 2 2 | -26947.07 | -26904.28 | 58.6 | 38.0 | 3.3 |
| 3 | 3 3 3 | -26954.73 | -26905.35 | 58.8 | 37.9 | 3.2 |
| 3 | 1 2 3 | -26989.94 | -26950.43 | 59.6 | 37.2 | 3.0 |
| 3 | 1 1 3 | -26992.01 | -26955.80 | 59.1 | 37.5 | 3.2 |
| 3 | 3 2 1 | -26953.60 | -26914.10 | 58.5 | 38.0 | 3.4 |
| 3 | 3 1 3 | -26960.51 | -26917.71 | 58.1 | 38.4 | 3.4 |
| 3 | 1 3 1 | -26997.96 | -26961.74 | 59.7 | 37.1 | 3.0 |
| 3 | 2 3 2 | -26951.90 | -26909.11 | 58.7 | 38.0 | 3.2 |
| 3 | 3 2 3 | -26951.24 | -26905.15 | 58.6 | 38.0 | 3.2 |
| 3 | 2 2 0 | -26956.83 | -26923.91 | 59.9 | 37.3 | 2.7 |

Table S3: Factor Loadings of 6 types of dietary patterns includes ‘Healthy’, ‘traditional’, ‘processed’, ‘confectionary’, ‘vegetarian’, and ‘high meat’ based on 55 food items across all time points (pregnancy, post pregnancy 4 years, 8/9 years, and 12/13 years) in ALSPAC Study

|  | **Pregnancy (32 weeks of gestation)** | | | | | **Post-pregnancy 4 years** | | | | **Post-pregnancy 8/9 years** | | | **Post-pregnancy 12/13 years** | | |
| --- | --- | --- | --- | --- | --- | --- | --- | --- | --- | --- | --- | --- | --- | --- | --- |
| **Food items** | Healthy | Traditional | Processed | Confec-tionary | Vege-tarian | Healthy | Processed | Confec-tionary | Vege-tarian | Healthy | Processed | High meat | Healthy | Processed | High meat |
| White bread | **-0.535** | **0.075** | **0.367** | 0.080 | -0.018 | -0.294 | **0.550** | 0.003 | -0.080 | -0.240 | 0.240 | **0.397** | **-0.348** | 0.272 | 0.249 |
| Non-white bread | **0.615** | -0.049 | -0.323 | -0.057 | 0.032 | **0.331** | **-0.546** | 0.077 | 0.124 | 0.235 | -0.097 | **-0.431** | **0.310** | -0.071 | -0.223 |
| Bran based cereal | **0.365** | 0.092 | -0.126 | -0.004 | 0.009 | 0.287 | -0.196 | 0.064 | -0.029 | 0.217 | -0.021 | -0.066 | 0.167 | 0.054 | -0.007 |
| Oat based cereals | 0.297 | 0.113 | -0.039 | 0.050 | 0.140 | 0.272 | -0.152 | 0.104 | 0.160 | 0.205 | 0.023 | -0.224 | 0.250 | -0.052 | -0.145 |
| Other breakfast cereals | -0.110 | -0.015 | 0.139 | 0.221 | -0.082 | -0.041 | 0.171 | 0.258 | -0.042 | -0.011 | 0.199 | 0.125 | -0.058 | 0.216 | 0.072 |
| Biscuits | 0.108 | 0.023 | -0.007 | **0.603** | -0.108 | -0.029 | 0.025 | **0.674** | -0.089 | -0.053 | **0.553** | -0.041 | -0.058 | **0.560** | -0.051 |
| Crispbread/crackers | 0.218 | 0.088 | -0.010 | 0.052 | 0.156 | 0.261 | -0.024 | -0.032 | 0.102 | 0.232 | -0.032 | -0.101 | 0.272 | -0.025 | -0.061 |
| Puddings | 0.265 | 0.064 | 0.124 | **0.389** | -0.112 | 0.101 | 0.121 | **0.475** | -0.017 | 0.119 | **0.480** | -0.015 | 0.140 | **0.519** | 0.023 |
| Yoghurts | NA | NA | NA | NA | NA | **0.353** | -0.165 | 0.260 | 0.041 | **0.334** | 0.054 | -0.140 | **0.371** | 0.066 | -0.016 |
| Ice cream | NA | NA | NA | NA | NA | 0.013 | 0.097 | **0.480** | -0.003 | 0.086 | **0.437** | -0.023 | 0.070 | **0.402** | -0.001 |
| Cakes/buns | 0.202 | 0.004 | 0.086 | 0.559 | -0.08 | 0.035 | 0.002 | **0.620** | -0.030 | 0.007 | **0.501** | -0.094 | 0.078 | **0.511** | -0.035 |
| Poultry | 0.270 | 0.223 | 0.121 | 0.023 | **-0.535** | **0.359** | 0.087 | 0.031 | **-0.502** | **0.333** | 0.026 | **0.406** | 0.263 | -0.064 | **0.602** |
| Red meats | 0.147 | 0.219 | 0.166 | 0.101 | **-0.596** | 0.207 | 0.168 | 0.090 | **-0.635** | 0.210 | 0.177 | **0.581** | 0.171 | 0.061 | **0.684** |
| Cold meats | NA | NA | NA | NA | NA | 0.092 | 0.073 | 0.186 | **-0.458** | 0.108 | 0.162 | **0.337** | 0.052 | 0.124 | **0.499** |
| Coated poultry products | NA | NA | NA | NA | NA | -0.011 | **0.434** | 0.137 | -0.119 | -0.013 | **0.393** | **0.317** | -0.096 | **0.344** | **0.305** |
| Meat pies | -0.105 | 0.032 | **0.538** | 0.087 | -0.118 | -0.165 | **0.520** | 0.149 | -0.064 | -0.125 | **0.470** | 0.304 | -0.185 | **0.411** | 0.270 |
| Offal | 0.087 | 0.091 | 0.248 | -0.066 | 0.087 | 0.108 | 0.177 | -0.035 | -0.069 | 0.142 | 0.101 | 0.197 | 0.067 | 0.074 | 0.137 |
| Sausages, burgers | -0.091 | -0.062 | **0.565** | 0.029 | -0.169 | -0.146 | **0.458** | 0.194 | -0.131 | -0.092 | **0.432** | 0.287 | -0.066 | 0.234 | **0.368** |
| Fried foods | -0.094 | 0.001 | **0.574** | 0.164 | -0.009 | NA | NA | NA | NA | NA | NA | NA | NA | NA | NA |
| Pizza | 0.233 | -0.105 | **0.349** | 0.104 | 0.105 | 0.014 | 0.277 | 0.250 | 0.274 | -0.037 | **0.495** | -0.116 | -0.005 | **0.462** | -0.047 |
| Breaded/battered white fish | NA | NA | NA | NA | NA | 0.096 | **0.413** | 0.173 | 0.073 | 0.120 | **0.369** | 0.139 | 0.032 | **0.347** | 0.193 |
| Fish* | **0.457** | 0.155 | 0.133 | -0.075 | -0.018 | **0.479** | -0.019 | -0.009 | 0.076 | 0.451 | 0.027 | -0.069 | **0.444** | -0.098 | 0.057 |
| Eggs | 0.278 | 0.090 | **0.403** | -0.027 | -0.016 | 0.232 | 0.201 | 0.111 | 0.037 | 0.206 | 0.249 | -0.009 | 0.235 | 0.163 | 0.146 |
| Cheese | **0.443** | 0.078 | 0.053 | 0.122 | 0.026 | 0.203 | -0.011 | 0.286 | 0.199 | 0.119 | **0.306** | -0.245 | 0.205 | 0.292 | -0.063 |
| Vegetarian pies | NA | **NA** | **NA** | NA | NA | 0.092 | 0.190 | 0.020 | **0.530** | 0.055 | **0.315** | **-0.366** | 0.101 | 0.188 | **-0.331** |
| Meat substitutes (soya, tofu etc) | 0.180 | 0.066 | 0.124 | -0.028 | **0.577** | 0.203 | **0.039** | -0.021 | **0.624** | 0.150 | 0.111 | **-0.498** | 0.215 | 0.079 | **-0.523** |
| Pulses | **0.356** | 0.146 | 0.006 | -0.055 | **0.565** | **0.365** | -0.116 | -0.044 | **0.489** | **0.319** | 0.018 | **-0.468** | **0.416** | -0.057 | **-0.370** |
| Nuts | 0.278 | 0.116 | 0.051 | 0.052 | **0.531** | 0.183 | -0.002 | 0.137 | **0.391** | 0.137 | 0.070 | **-0.344** | 0.287 | -0.059 | -0.269 |
| Chips | -0.255 | -0.057 | **0.561** | 0.235 | -0.036 | -0.148 | **0.566** | 0.284 | 0.089 | -0.127 | **0.606** | 0.199 | -0.200 | **0.463** | 0.179 |
| Roasted potatoes | -0.271 | 0.225 | **0.388** | 0.154 | -0.165 | 0.036 | **0.467** | 0.117 | -0.205 | 0.051 | **0.340** | **0.378** | -0.050 | **0.432** | 0.256 |
| Potatoes (not chips) | 0.254 | **0.321** | 0.104 | 0.070 | -0.219 | **0.387** | 0.136 | 0.058 | -0.130 | **0.413** | 0.131 | 0.103 | **0.348** | 0.214 | 0.226 |
| Pasta | **0.578** | 0.045 | 0.136 | -0.070 | 0.121 | **0.422** | -0.113 | 0.039 | 0.215 | **0.411** | 0.033 | -0.223 | **0.410** | 0.062 | 0.018 |
| Rice | **0.543** | 0.078 | 0.125 | -0.120 | 0.063 | **0.421** | -0.040 | 0.015 | 0.113 | **0.414** | 0.051 | -0.115 | **0.376** | 0.054 | 0.098 |
| Baked beans* | 0.004 | 0.049 | 0.413 | 0.081 | 0.045 | 0.113 | **0.473** | 0.135 | 0.112 | 0.134 | **0.384** | 0.140 | 0.045 | **0.457** | 0.107 |
| Leafy green vegetables | 0.045 | **0.809** | 0.011 | -0.015 | 0.041 | **0.667** | 0.124 | -0.150 | -0.130 | **0.661** | -0.050 | 0.101 | **0.531** | -0.162 | 0.011 |
| Other green vegetables | 0.147 | **0.799** | -0.043 | -0.004 | 0.054 | **0.697** | 0.109 | -0.149 | -0.128 | **0.696** | -0.059 | 0.077 | **0.473** | -0.160 | 0.046 |
| Carrots | 0.178 | **0.704** | -0.020 | 0.023 | 0.008 | **0.660** | 0.095 | -0.053 | -0.139 | **0.736** | -0.030 | 0.113 | **0.573** | 0.068 | 0.154 |
| Other root vegetables | 0.084 | **0.606** | 0.018 | 0.003 | 0.106 | **0.549** | 0.162 | -0.131 | -0.053 | **0.772** | -0.023 | 0.134 | **0.508** | 0.031 | 0.134 |
| Peas | 0.174 | **0.352** | 0.190 | 0.063 | -0.104 | 0.297 | **0.344** | 0.060 | -0.039 | **0.347** | 0.245 | 0.161 | **0.345** | 0.283 | 0.169 |
| Sweetcorn | NA | NA | NA | NA | NA | **0.412** | 0.216 | -0.040 | 0.003 | **0.368** | 0.140 | -0.002 | **0.396** | 0.209 | 0.054 |
| Salad | **0.420** | 0.212 | -0.078 | -0.022 | 0.100 | **0.395** | 0.146 | -0.011 | 0.157 | **0.529** | -0.066 | -0.228 | **0.546** | -0.132 | -0.064 |
| Fresh fruits | **0.518** | 0.182 | -0.229 | 0.090 | 0.005 | **0.491** | -0.247 | 0.130 | 0.039 | **0.499** | -0.036 | **-0.219** | **0.426** | -0.139 | -0.118 |
| Fruit juice | **0.488** | 0.079 | -0.090 | 0.085 | 0.057 | 0.252 | -0.209 | 0.242 | 0.091 | 0.230 | 0.133 | -0.214 | 0.249 | 0.094 | -0.128 |
| Fizzy drinks | NA | NA | NA | NA | NA | -0.052 | 0.184 | 0.219 | -0.109 | -0.047 | 0.232 | 0.231 | -0.053 | 0.191 | 0.077 |
| Cola drinks | -0.209 | -0.081 | 0.221 | 0.142 | 0.051 | NA | NA | NA | NA | NA | NA | NA | NA | NA | NA |
| Tea | -0.100 | 0.078 | 0.156 | 0.029 | -0.037 | 0.014 | 0.108 | 0.003 | 0.041 | NA | NA | NA | NA | NA | NA |
| Coffee | -0.161 | 0.053 | 0.105 | 0.002 | -0.037 | 0.002 | 0.008 | -0.084 | -0.048 | NA | NA | NA | NA | NA | NA |
| Herbal tea | 0.186 | 0.068 | -0.085 | -0.057 | **0.302** | 0.155 | -0.123 | -0.024 | 0.205 | -0.227 | 0.079 | **0.381** | NA | NA | NA |
| Water | NA | NA | NA | NA | NA | 0.232 | -0.103 | 0.128 | 0.066 | 0.273 | 0 | -0.187 | **0.360** | -0.114 | -0.095 |
| Flavoured milk drinks | NA | NA | NA | NA | NA | 0.087 | 0.130 | 0.165 | 0.070 | 0.128 | 0.221 | -0.013 | 0.067 | 0.190 | -0.039 |
| Squash | NA | NA | NA | NA | NA | 0.031 | 0.134 | **0.323** | -0.078 | 0.086 | 0.256 | 0.091 | 0.032 | 0.256 | 0.090 |
| Sweets | -0.098 | 0.071 | 0.069 | **0.514** | 0.061 | -0.044 | 0.092 | **0.371** | -0.077 | -0.019 | **0.325** | 0.009 | -0.058 | **0.378** | 0.003 |
| Chocolate | 0.000 | 0.022 | 0.036 | **0.717** | 0.058 | -0.053 | 0.019 | **0.578** | -0.026 | -0.044 | **0.455** | -0.095 | -0.027 | **0.445** | -0.103 |
| Chocolate bar | -0.085 | -0.020 | 0.096 | **0.749** | 0.021 | NA | NA | NA | NA | NA | NA | NA | NA | NA | NA |
| Crisps | -0.101 | -0.041 | 0.292 | **0.381** | 0.004 | -0.077 | 0.177 | **0.474** | -0.002 | -0.106 | **0.434** | 0.050 | -0.121 | **0.415** | 0.016 |

* Baked beans: At 4-years post-pregnancy tinned pasta included.

* Chocolate bar: Chocolate bar combined with chocolate at 4-, 8/9-, 12/13 years post-pregnancy.

* Factor loadings ≥ 0·3 are shown in bold

Table S4: The distribution of maternal characteristics of complete cohort (5,336) at the baseline by “healthy” DP trajectories in ALSPAC

| **Maternal characteristics** | **Lower healthy trajectory (n=1,971)** | | **Moderately healthy trajectory (n=2,918)** | **Higher healthy trajectory (n=447)** | | **p-value** |
| --- | --- | --- | --- | --- | --- | --- |
| **Maternal age** |  |  | | |  |  |
| <= 20 years | 78 (4.0) | 32 (1.1) | | | 6 (1.3) | <0.001 |
| 21-30 years | 1310 (66.4) | 1635 (56.0) | | | 222 (49.7) |  |
| >= 30 years | 583 (29.6) | 1251 (42.9) | | | 219 (49.0) |  |
| **Maternal education** | | | | | | |
| CSE/none/Vocational | 539 (28.9) | 293 (10.2) | | | 44 (10.1) | <0.001 |
| O level | 841 (45.0) | 904 (31.5) | | | 117 (26.8) |  |
| A level & higher | 488 (26.1) | 1671 (58.3) | | | 275 (63.1) |  |
| **Employment Status** | | | | | | |
| Unemployment | 631 (34.3) | 794 (29.1) | | | 155 (37.5) | <0.001 |
| Employment | 1207 (65.7) | 1936 (70.9) | | | 258 (62.5) |  |
| **Marital status** |  |  | | |  |  |
| Never married | 290 (14.8) | 274 (9.5) | | | 53 (12.0) | <0.001 |
| Ever Married | 1663 (85.2) | 2626 (90.5) | | | 390 (88.0) |  |
| **Social class** |  |  | | |  |  |
| High social class | 407 (22.8) | 1245 (47.0) | | | 198 (49.2) | <0.001 |
| Middle social class | 1013 (56.7) | 1074 (40.5) | | | 142 (35.2) |  |
| Low social class | 365 (20.5) | 333 (12.5) | | | 63 (15.6) |  |
| **Ethnic group** |  |  | | |  |  |
| White | 1940 (98.8) | 2871 (98.7) | | | 436 (98.0) | 0.35 |
| Non-white | 23 (1.2) | 37 (1.3) | | | 9 (2.0) |  |
| **Smoking Status*** |  |  | | |  |  |
| Non-smoker | 1449 (78.1) | 2517 (91.5) | | | 396 (93.2) | <0.001 |
| Light smoker | 149 (8.0) | 132 (4.8) | | | 21 (4.9) |  |
| Heavy smoker | 257 (13.9) | 103 (3.7) | | | 8 (1.9) |  |
| **Alcohol consumption** |  |  | | |  |  |
| Never/< 1 glass/week | 981 (50.2) | 1093 (37.6) | | | 174 (39.6) | <0.001 |
| 1+ glasses/week | 788 (40.3) | 1422 (49.0) | | | 207 (47.0) |  |
| 1+ glasses/day | 185 (9.5) | 388 (13.4) | | | 59 (13.4) |  |
| **Parity (number of previous pregnancies)** | | | | | | |
| None | 906 (47.5) | 1388 (48.8) | | | 183 (42.2) | <0.01 |
| One | 664 (34.8) | 1044 (36.7) | | | 171 (39.4) |  |
| Two | 256 (13.4) | 312 (11.0) | | | 65 (15.0) |  |
| Three | 83 (4.3) | 101 (3.5) | | | 15 (3.4) |  |
| **Pre-pregnancy BMI** | | | | | | |
| Normal | 1437 (79.3) | 2275 (82.1) | | | 344 (80.6) | 0.10 |
| Overweight | 278 (15.3) | 375 (13.6) | | | 67 (15.7) |  |
| Obesity | 98 (5.4) | 120 (4.3) | | | 16 (3.7) |  |

*Data are presented as n (%) of total by three trajectories in ALSPAC study.

*Smoking status: Non-smoker (No), Light smoker (Yes-1 to 9 cig/day) and Heavy smoker (Yes-10+ cig/day).

Table S5: The distribution of maternal characteristics of complete cohort (5,336) at the baseline by “processed” DP trajectories in ALSPAC

| **Maternal characteristics** | **Lower processed trajectory (n=3,171)** | **Moderately processed trajectory (n=2,011)** | **Higher processed trajectory (154)** | **p-value** |
| --- | --- | --- | --- | --- |
| **Maternal age** |  |  |  |  |
| <= 20 years | 23 (0.7) | 82 (4.1) | 11 (7.2) | <0.001 |
| 21-30 years | 1698 (53.6) | 1361 (67.7) | 108 (70.1) |  |
| >= 30 years | 1450 (45.7) | 568 (28.2) | 35 (22.7) |  |
| **Maternal education** |  |  |  |  |
| CSE/none/Vocational | 382 (12.3) | 440 (22.7) | 54 (38.8) | <0.001 |
| O level | 925 (29.9) | 877 (45.3) | 60 (43.2) |  |
| A level & higher | 1790 (57.8) | 619 (32.0) | 25 (18.0) |  |
| **Employment Status** |  |  |  |  |
| Unemployment | 853 (28.7) | 662 (35.4) | 65 (46.8) | <0.001 |
| Employment | 2116 (71.3) | 1214 (64.6) | 74 (53.2) |  |
| **Marital status** |  |  |  |  |
| Never married | 326 (10.3) | 258 (13.0) | 33 (21.6) | <0.001 |
| Ever Married | 2827 (89.7) | 1732 (87.0) | 120 (78.4) |  |
| **Social class** |  |  |  |  |
| High social class | 1347 (46.6) | 479 (26.4) | 24 (18.2) | <0.001 |
| Middle social class | 1174 (40.6) | 980 (54.0) | 75 (56.8) |  |
| Low social class | 372 (12.8) | 356 (19.6) | 33 (25.0) |  |
| **Ethnic group** |  |  |  |  |
| White | 3125 (98.9) | 1974 (98.5) | 148 (96.7) | <0.05 |
| Non-white | 34 (1.1) | 30 (1.5) | 5 (3.3) |  |
| **Smoking Status*** |  |  |  |  |
| Non-smoker | 2702 (89.9) | 1555 (82.5) | 105 (73.9) | <0.001 |
| Light smoker | 150 (5.0) | 138 (7.3) | 14 (9.9) |  |
| Heavy smoker | 153 (5.1) | 192 (10.2) | 23 (16.2) |  |
| **Alcohol consumption** |  |  |  |  |
| Never/< 1 glass/week | 1202 (38.1) | 970 (48.7) | 76 (50.0) | <0.001 |
| 1+ glasses/week | 1504 (47.7) | 853 (42.9) | 60 (39.5) |  |
| 1+ glasses/day | 449 (14.2) | 167 (8.4) | 16 (10.5) |  |
| **Number of previous pregnancies** |  |  |  |  |
| None | 1506 (48.8) | 907 (46.5) | 64(43.0) | 0.33 |
| One | 1111 (36.0) | 714 (36.6) | 54(36.2) |  |
| Two | 355 (11.5) | 255 (13.7) | 23(15.4) |  |
| Three | 116 (3.7) | 75 (3.8) | 8(5.4) |  |
| **Pre-pregnancy BMI** |  |  |  |  |
| Normal | 2469 (82.2) | 1479 (79.3) | 108 (76.1) | <0.01 |
| Overweight | 422 (14.1) | 274 (14.7) | 24 (16.9) |  |
| Obesity | 111 (3.7) | 113 (6.0) | 10 (7.0) |  |

*Data are presented as n (%) of total by three trajectories in ALSPAC study.

*Smoking status: Non-smoker (No), Light smoker (Yes-1 to 9 cig/day) and Heavy smoker (Yes-10+ cig/day).

Table S5: Distribution of missing data per variable in baseline characteristics of women during pregnancy (complete case, n=5,336) in ALSPAC

| **Covariates and outcome data used in this study** | **Number of participants** | **Missing observation n (%)** |
| --- | --- | --- |
| Maternal age | 5,336 | 0 (0%) |
| Maternal education | 5,172 | 164 (3.1%) |
| Employment Status | 4,984 | 352 (6.6%) |
| Marital Status | 5,296 | 40 (0.7%) |
| Social class | 4,840 | 496 (9.3%) |
| Ethnic group | 5,316 | 20 (0.4%) |
| Smoking Status | 5,032 | 304 (5.7%) |
| Regular alcohol drinking pattern | 5,297 | 39 (0.7%) |
| Parity | 5,188 | 148 (2.7%) |
| Pre-pregnancy BMI | 5,010 | 326 (6.1%) |
| Dietary patterns at 32 weeks of pregnancy |  |  |
| Healthy DP | 5,336 | 0 (0%) |
| Traditional DP | 5,336 | 0 (0%) |
| Processed DP | 5,336 | 0 (0%) |
| Confectionary DP | 5,336 | 0 (0%) |
| Vegetarian DP | 5,336 | 0 (0%) |
| Dietary patterns at 4 years of post-pregnancy | | |
| Healthy DP | 5,336 | 0 (0%) |
| Processed DP | 5,336 | 0 (0%) |
| Confectionary DP | 5,336 | 0 (0%) |
| Vegetarian DP | 5,336 | 0 (0%) |
| Dietary patterns at 8/9 years of post-pregnancy | | |
| Healthy DP | 5,336 | 0 (0%) |
| Processed DP | 5,336 | 0 (0%) |
| High meat DP | 5,336 | 0 (0%) |
| Dietary patterns at 12 years of post-pregnancy | | |
| Healthy DP | 5,336 | 0 (0%) |
| Processed DP | 5,336 | 0 (0%) |
| High meat DP | 5,336 | 0 (0%) |
